# Supplementary material for: Solvent-free microwave synthesis of ultra-small Ru-Mo2C@CNT with strong metal-support interaction for industrial hydrogen evolution
Source: Nat Commun. 2021 Jun 29;12:4018. doi: 10.1038/s41467-021-24322-2 (PMC8242096; doi:10.1038/s41467-021-24322-2)
Supplement: Supplementary file 2 — Supplementary Info [file 41467_2021_24322_MOESM2_ESM.pdf]

Supporting Information for

Solvent-free microwave synthesis of ultra-small Ru-Mo_2C@CNT with strong metal-support interaction for industrial hydrogen evolution

Xueke Wu[1], Zuochao Wang[1], Dan Zhang[1,2], Yingnan Qin[1], Minghui Wang[1], Yi Han[1], Tianrong Zhan[1], Bo Yang[2], Shaoxiang Li[2], Jianping Lai[1,] and Lei Wang[1,2,]**

[1]Key Laboratory of Eco-chemical Engineering, Key Laboratory of Optic-electric Sensing and Analytical Chemistry of Life Science, Taishan Scholar Advantage and Characteristic Discipline Team of Eco Chemical Process and Technology, College of Chemistry and Molecular Engineering, Qingdao University of Science and Technology, Qingdao 266042, P. R. China

E-mail: inorchemwl@126.com; jplai@qust.edu.cn

[2]Shandong Engineering Research Center for Marine Environment Corrosion and Safety Protection, College of Environment and Safety Engineering, Qingdao University of Science and Technology, Qingdao 266042, P. R. China

Figures

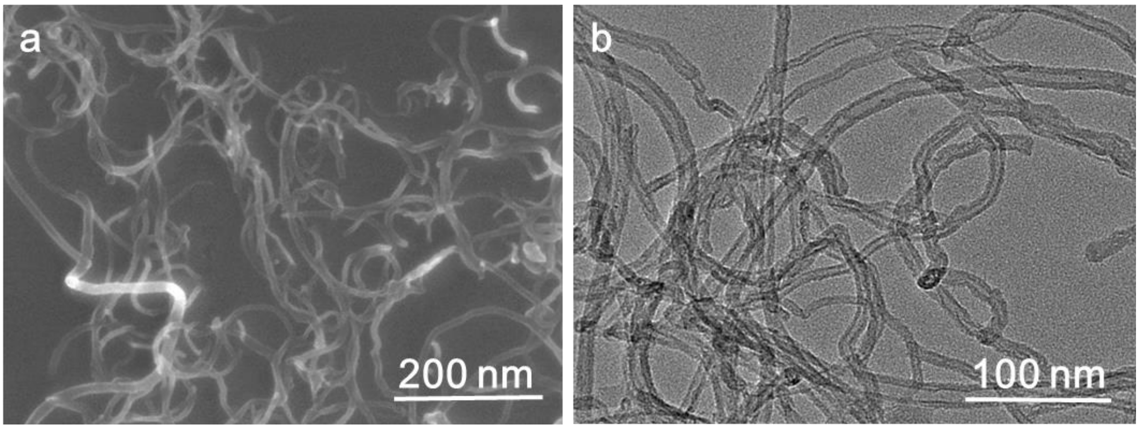

Supplementary Figure 1. (a) SEM and (b) TEM images of MWCNT.

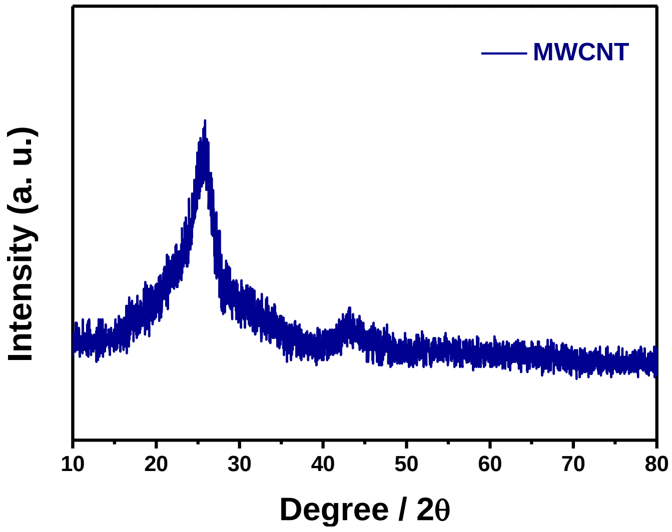

Supplementary Figure 2. XRD pattern of MWCNT.

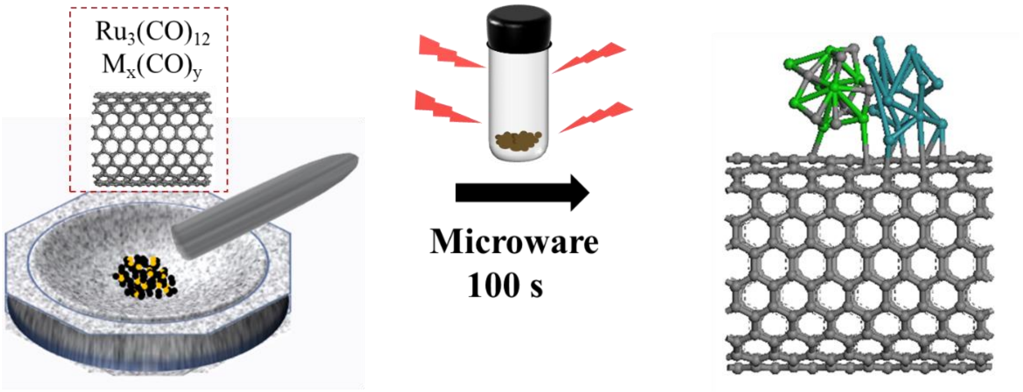

Supplementary Figure 3. Schematic diagram illustrating the synthetic procedure of Ru-M_xC@CNT.

(green: Mo; blue: Ru; gray: C)

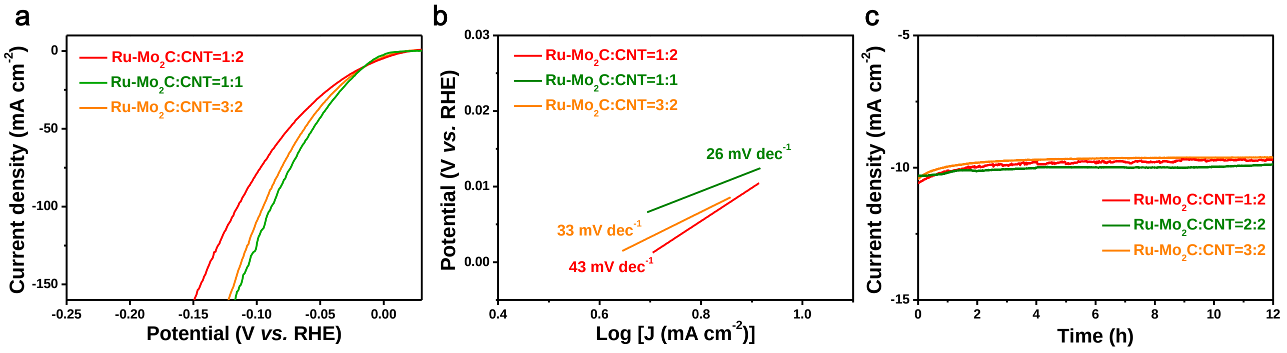

Supplementary Figure 4. (a) Polarization curves of the Ru-Mo_2C@MWCNT catalysts prepared under different Ru-Mo_2C@CNT ratios of 1:2, 1:1 and 3:2 in 1.0 M KOH solution. (b) Corresponding tafel slopes in 1.0 M KOH solution. (c) Current-time (i-t) stability curves up to 12 h duration of different Ru-Mo_2C@CNT ratios of 1:2, 1:1 and 3:2 were recorded in 1.0 M KOH solutions.

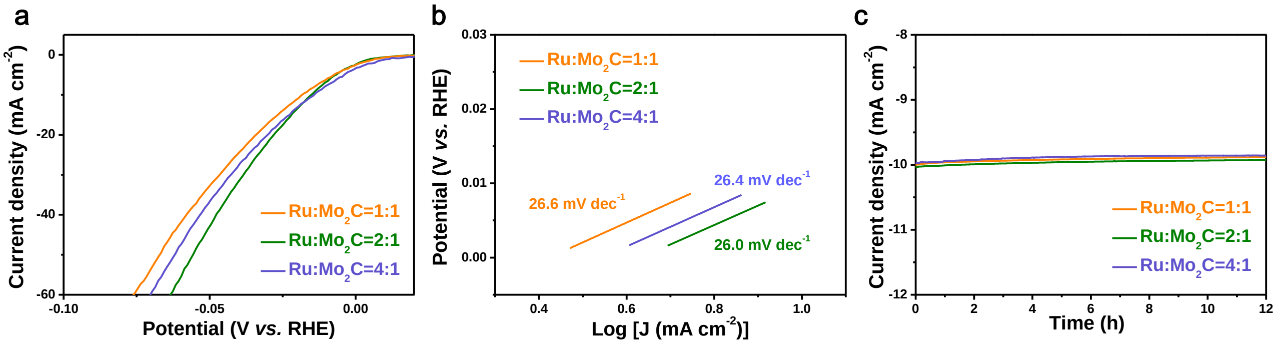

Supplementary Figure 5. (a) Polarization curves of the Ru-Mo_2C@MWCNT catalysts prepared under different Ru: Mo_2C ratios of 1:1, 2:1 and 4:1 in 1.0 M KOH solution. (b) Corresponding Tafel slopes in 1.0 M KOH solution. (c) Current-time (i-t) stability curves up to 12 h duration of different Ru: Mo_2C ratios of 1:1, 2:1 and 4:1 were recorded in 1.0 M KOH solutions.

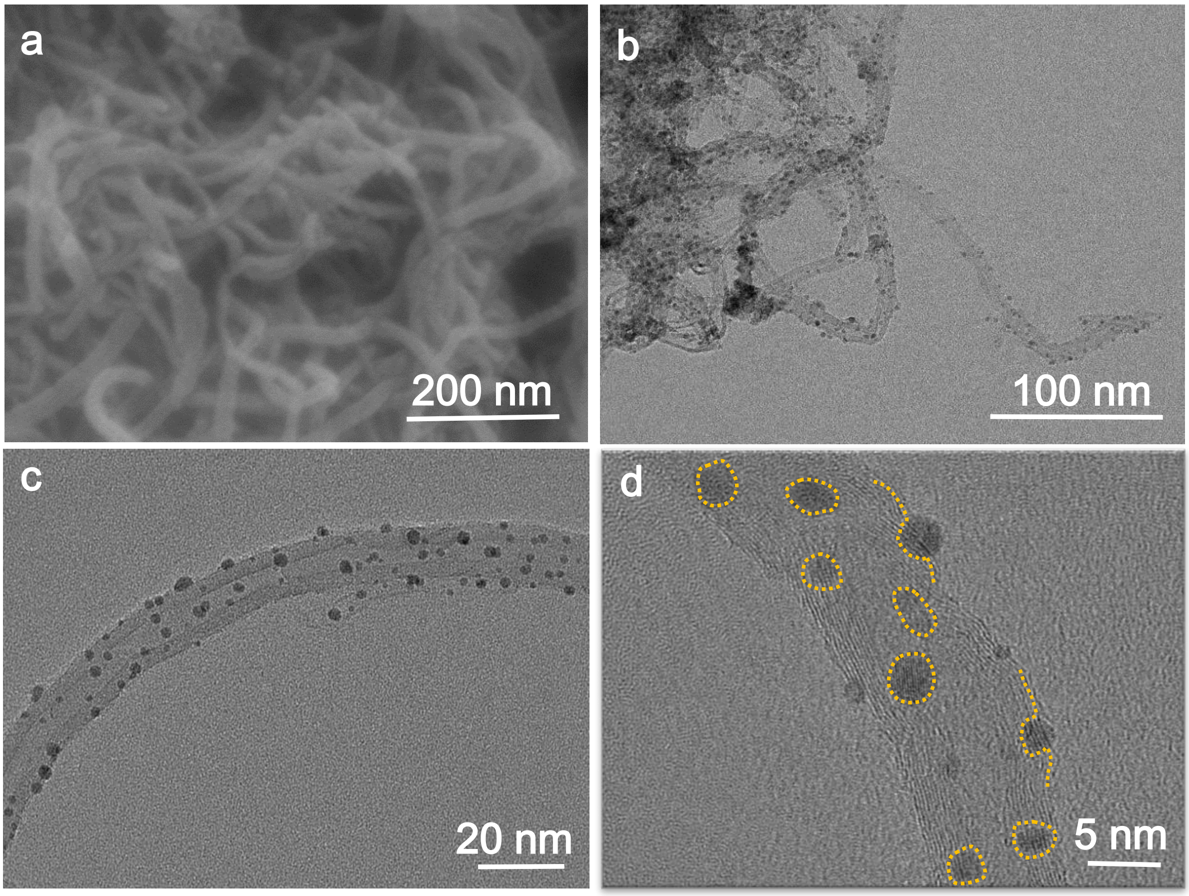

Supplementary Figure 6. (a) SEM, (b, c) TEM and (d) HRTEM images of Ru-Mo_2C@CNT.

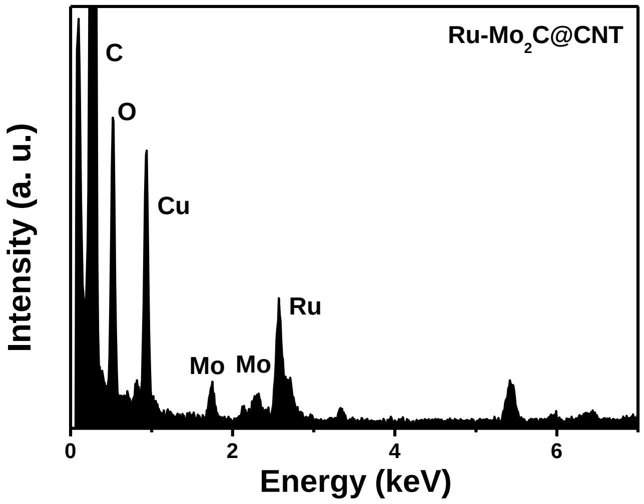

Supplementary Figure 7. EDX spectrum of Ru-Mo_2C@CNT.

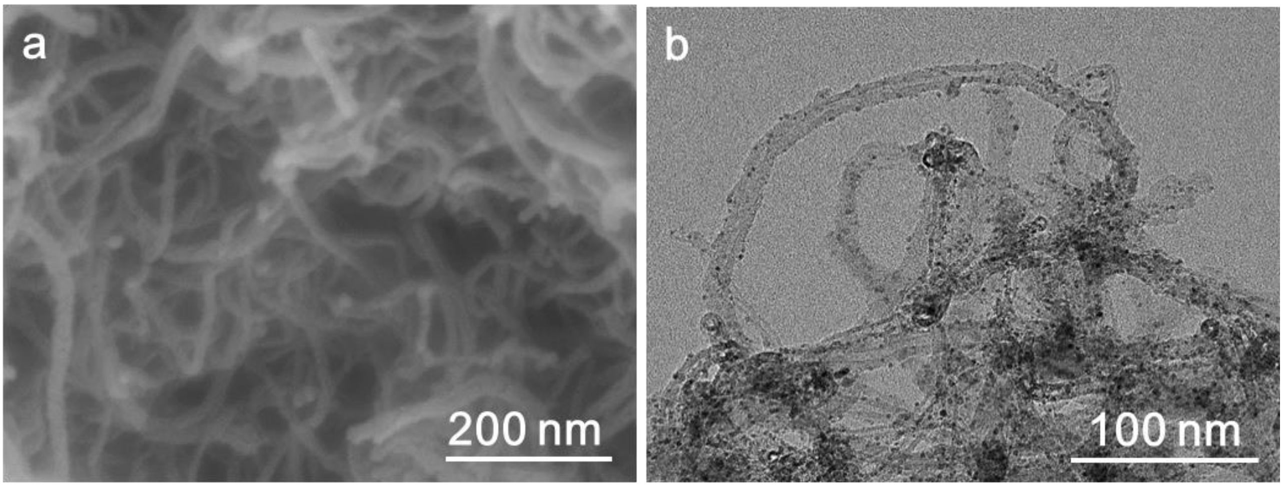

Supplementary Figure 8. (a) SEM and (b) TEM images of Ru@CNT.

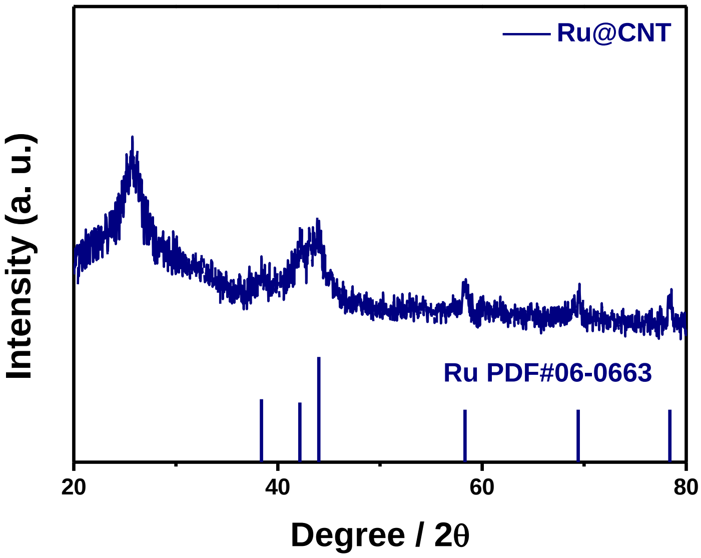

Supplementary Figure 9. XRD pattern of Ru@CNT.

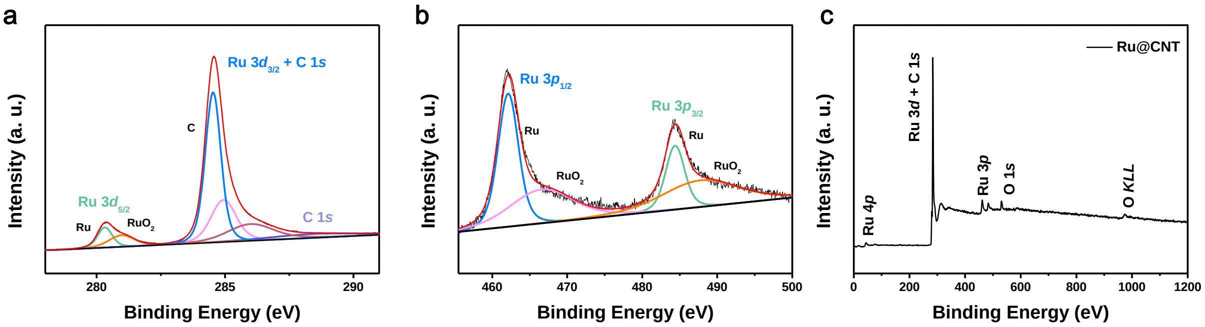

Supplementary Figure 10. XPS spectra of Ru@CNT (a) Ru 3*d* + C1*s*, (b) Ru 3*p* and (c) survey.

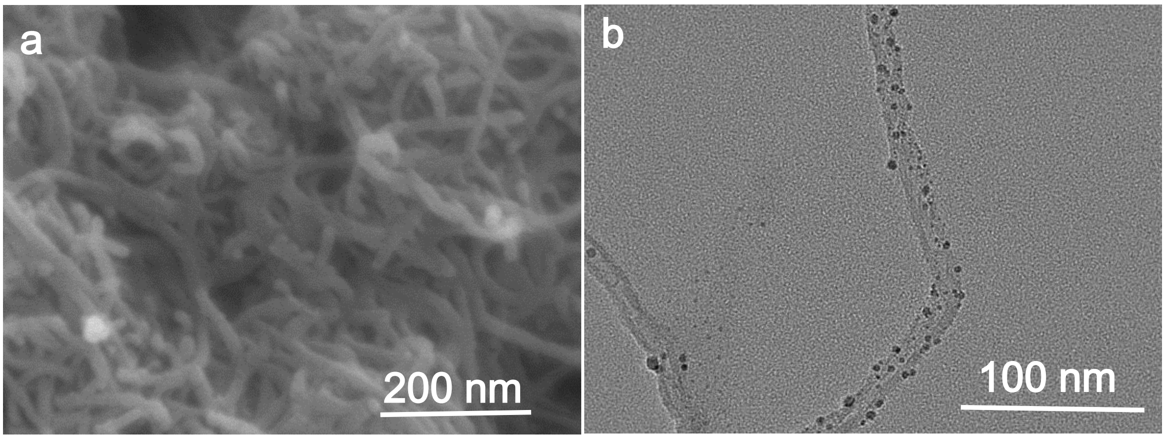

Supplementary Figure 11. (a) SEM and (b) TEM images of Mo_2C@CNT.

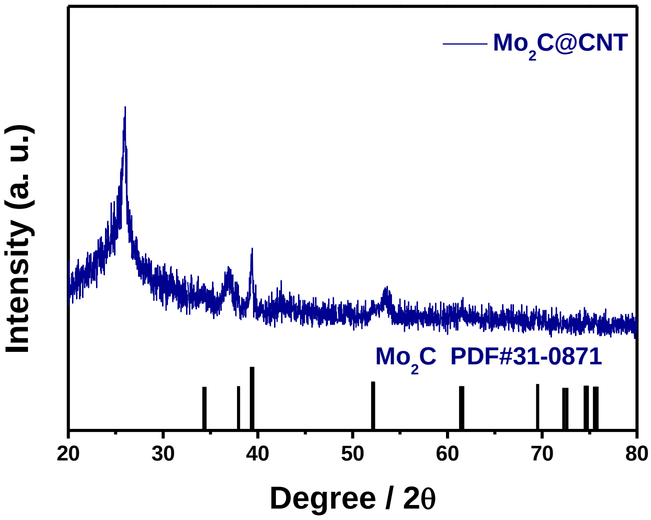

Supplementary Figure 12. XRD pattern of Mo_2C@CNT.

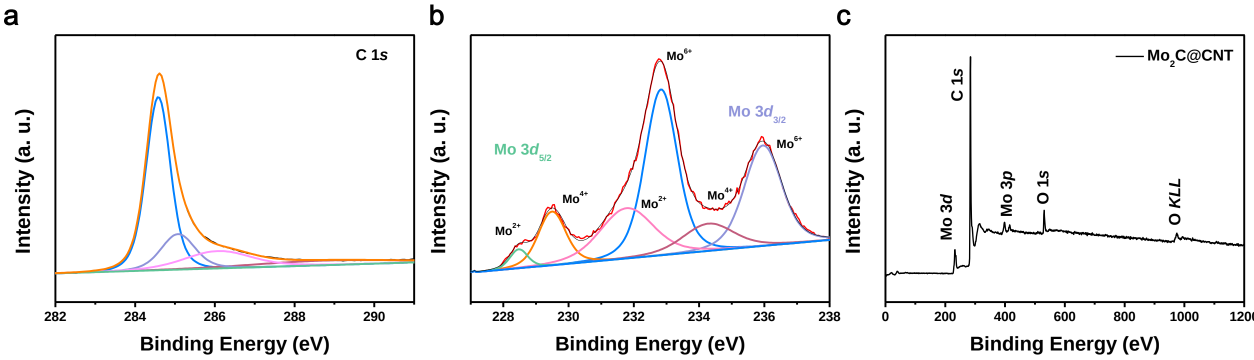

Supplementary Figure 13. XPS spectra of Mo_2C@CNT: (a) C 1*s*, (b) Mo 3*d* and (c) survey.

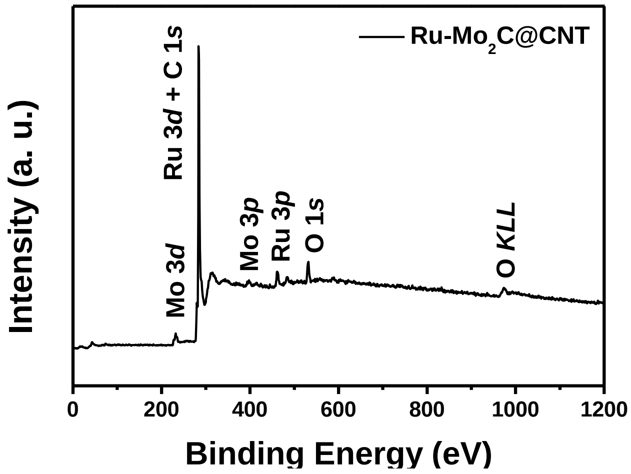

Supplementary Figure 14. XPS spectrum of Ru-Mo_2C@CNT.

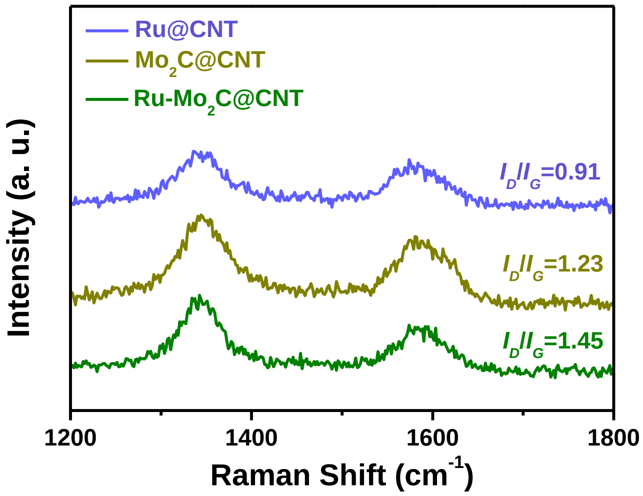

Supplementary Figure 15. Raman spectra of Ru@CNT, Mo_2C@CNT and Ru-Mo_2C@CNT catalysts.

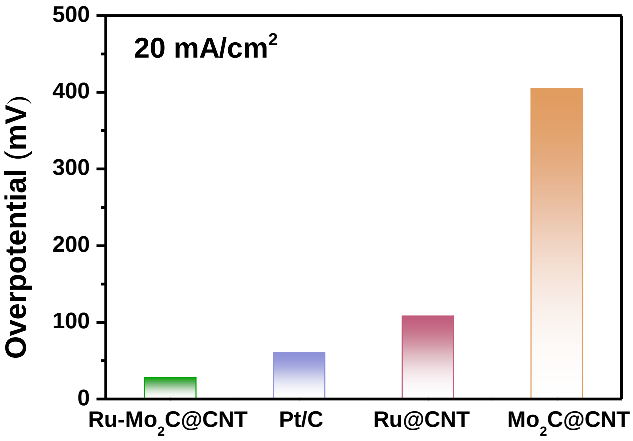

Supplementary Figure 16. Comparison of overpotential changes at 20 mA cm^{-2} of Ru-Mo_2C@CNT, Pt/C, Ru@CNT and Mo_2C@CNT catalysts.

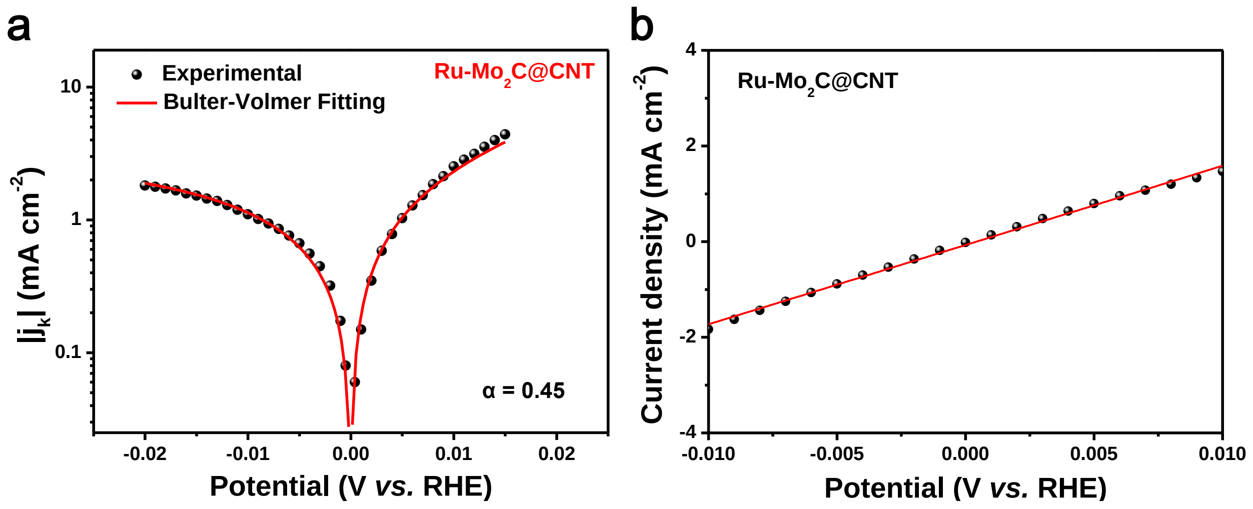

Supplementary Figure 17. (a) HOR/HER Tafel plots of the kinetic current density on Ru-Mo_2C@CNT in H_2-saturated 1.0 M KOH. The solid line indicate the Butler-Volmer fitting. (b) Micropolarization regions (-10 mV to 10 mV) of Ru-Mo_2C@CNT. In the Micropolarization region, Bulter-Volmer equation can be simplified to $j_0 = \frac{j}{\eta}\frac{RT}{F}$. where j is the measured current density, η is the overpotential, R is the universal gas constant, T is the temperature, and F is Faraday's constant. Therefore, the exchange current density (j_0) can be obtained from the slope of the linear fitting of j-η curve in micropolarization regions.

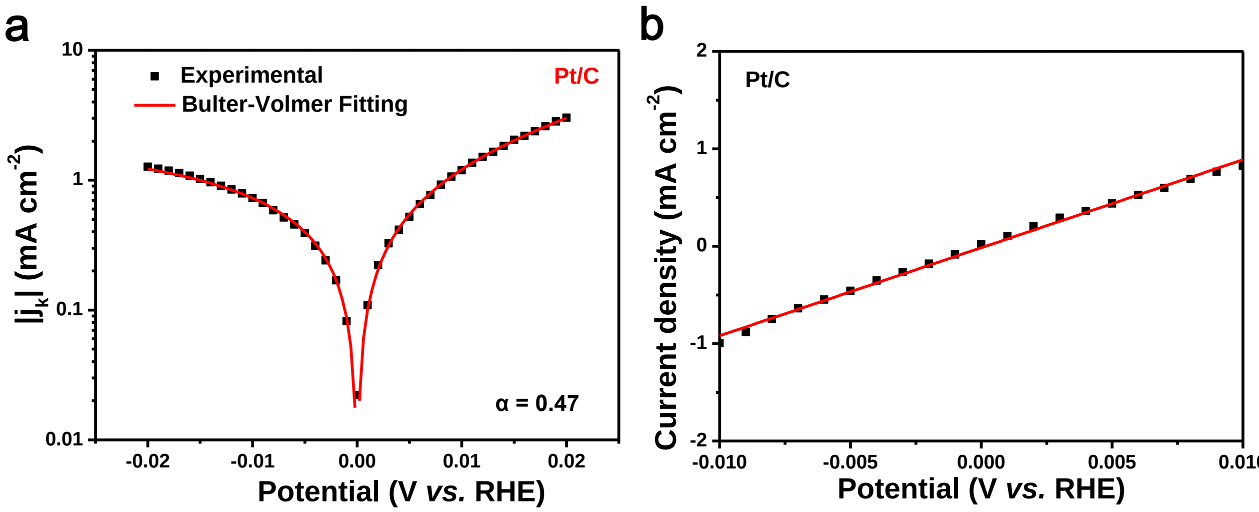

Supplementary Figure 18. (a) HOR/HER Tafel plots of the kinetic current density on Pt/C in H_2-saturated 1.0 M KOH. The solid line indicate the Butler-Volmer fitting. (b) Micropolarization regions (-10 mV to 10 mV) of Ru-Mo_2C@CNT.

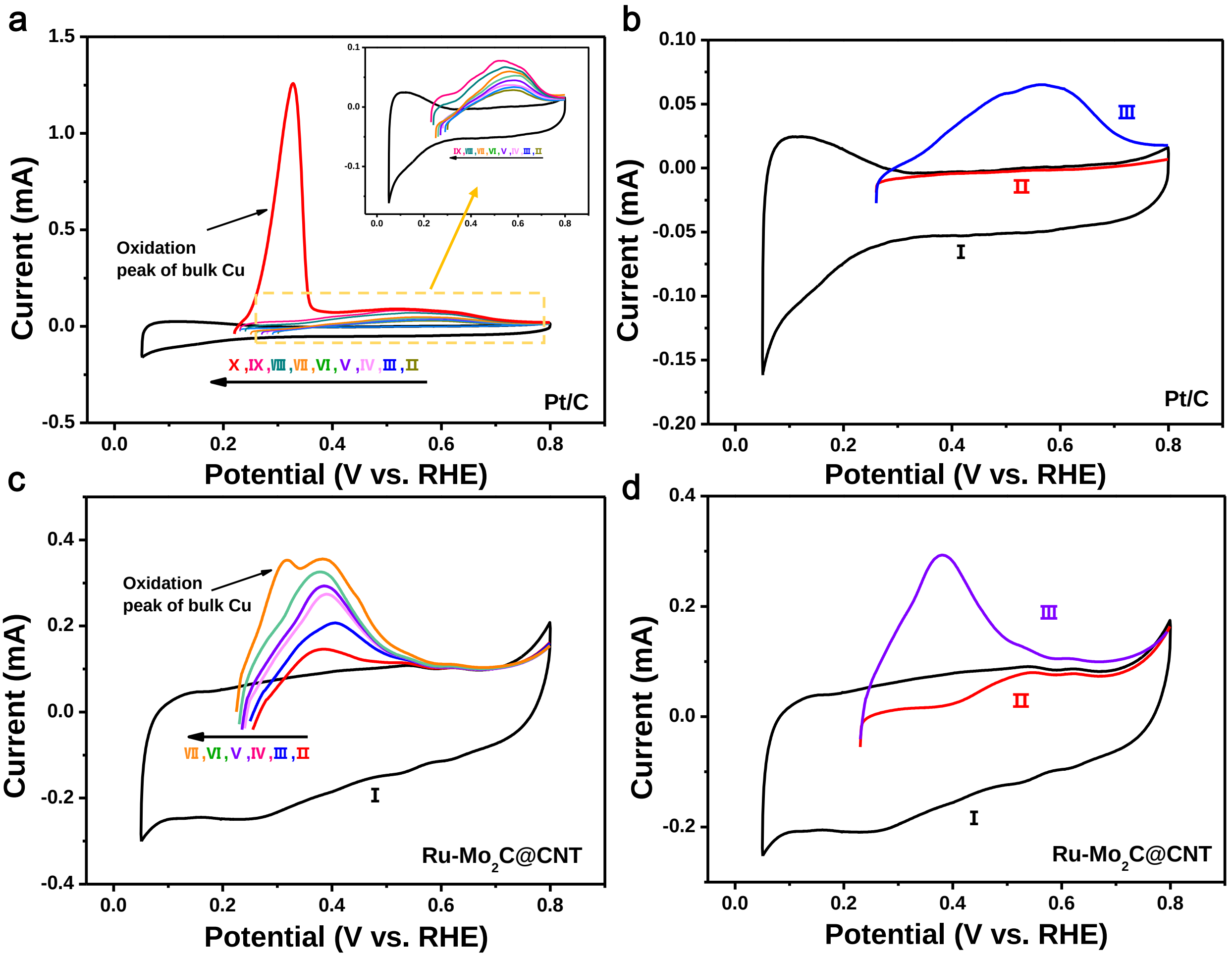

Supplementary Figure 19. (a) Copper UPD in 0.5 M H_2SO_4 in the (I) absence and (II- X) presence of 5 mM $CuSO_4$ on Pt/C. For II-X, the electrode was polarized at 0.3V, 0.29V, 0.28V, 0.27V, 0.26V, 0.25V, 0.24V, 0.23V and 0.22V for 100 s to form the UPD layers, respectively. (b) Copper UPD in 0.5 M H_2SO_4 in the (I, II) absence and (III) presence of 5 mM $CuSO_4$ on Pt/C. For II and III, the electrode was polarized at 0.26 V for 100 s to form the UPD layer. (c) Copper UPD in 0.5 M H_2SO_4 in the (I) absence and (II-VII) presence of 5 mM $CuSO_4$ on Ru-Mo_2C@CNT. For II-VII, the electrode was polarized at 0.26V, 0.25V, 0.24V, 0.235V, 0.23V and 0.225V for 100 s to form the UPD layers, respectively. Copper UPD in 0.5 M H_2SO_4 in the absence and presence of 5 mM $CuSO_4$ on Ru-Mo_2C@CNT. The electrode was polarized at 0.23 V for 100 s to form the UPD layer. (d) Copper UPD in 0.5 M H_2SO_4 in the (I, II) absence and (III) presence of 5 mM $CuSO_4$ on Ru-Mo_2C@CNT. For II and III, the electrode was polarized at 0.23 V for 100 s to form the UPD layer.

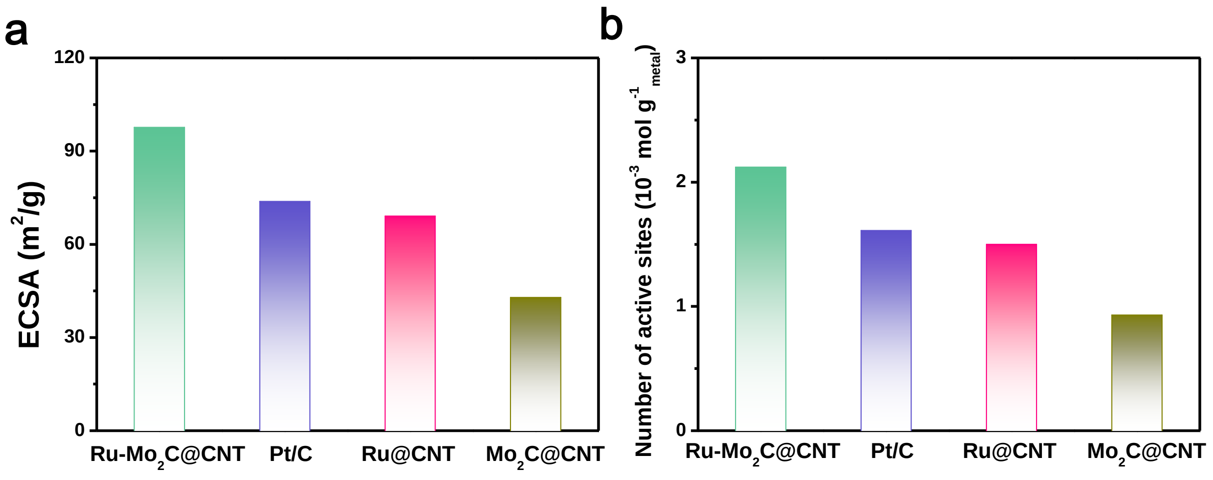

Supplementary Figure 20. (a) Estimation of the ECSA and (b) active sites using the Cu-UPD method of Ru-Mo_2C@CNT, Pt/C, Ru@CNT and Mo_2C@CNT.

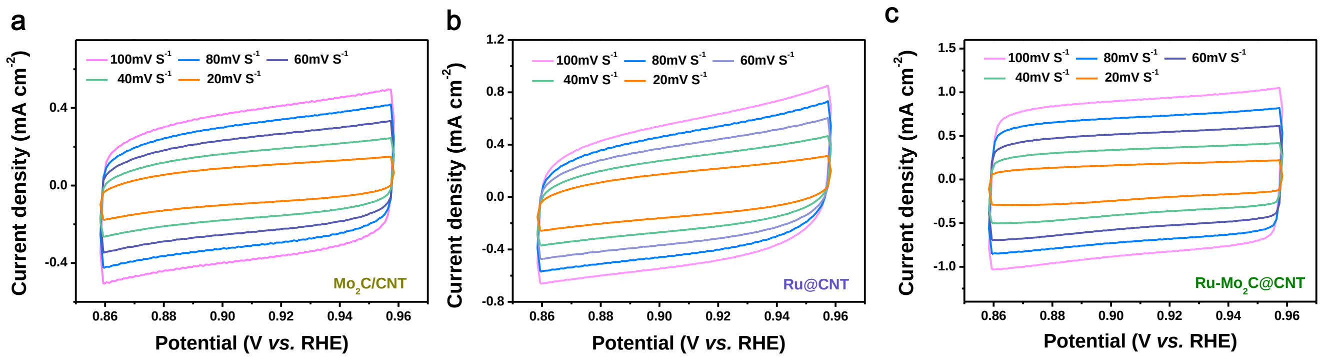

Supplementary Figure 21. CV curves measured at different scan rates from 20 to 100 mV s^{-1} in 1.0 M KOH for (a) Mo_2C@CNT, (b) Ru@CNT and (c) Ru-Mo_2C@CNT.

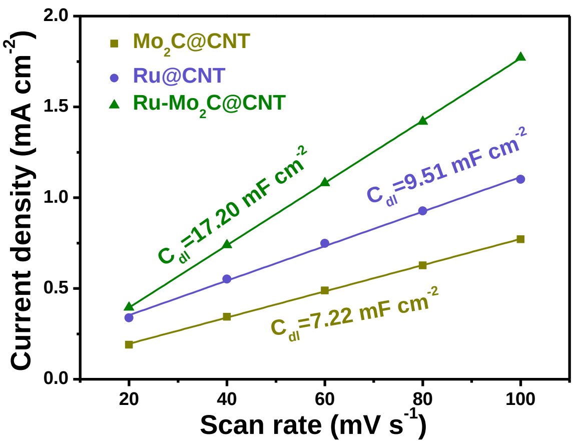

Supplementary Figure 22. Capacitive current at middle potential of CV curves as function of scan rates for Mo_2C@CNT, Ru@CNT and Ru-Mo_2C@CNT.

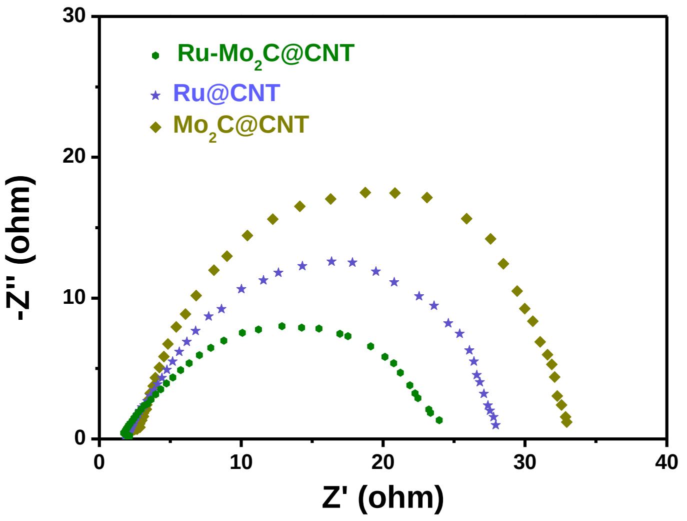

Supplementary Figure 23. EIS Nyquist plots of Mo_2C@CNT, Ru@CNT and Ru-Mo_2C@CNT.

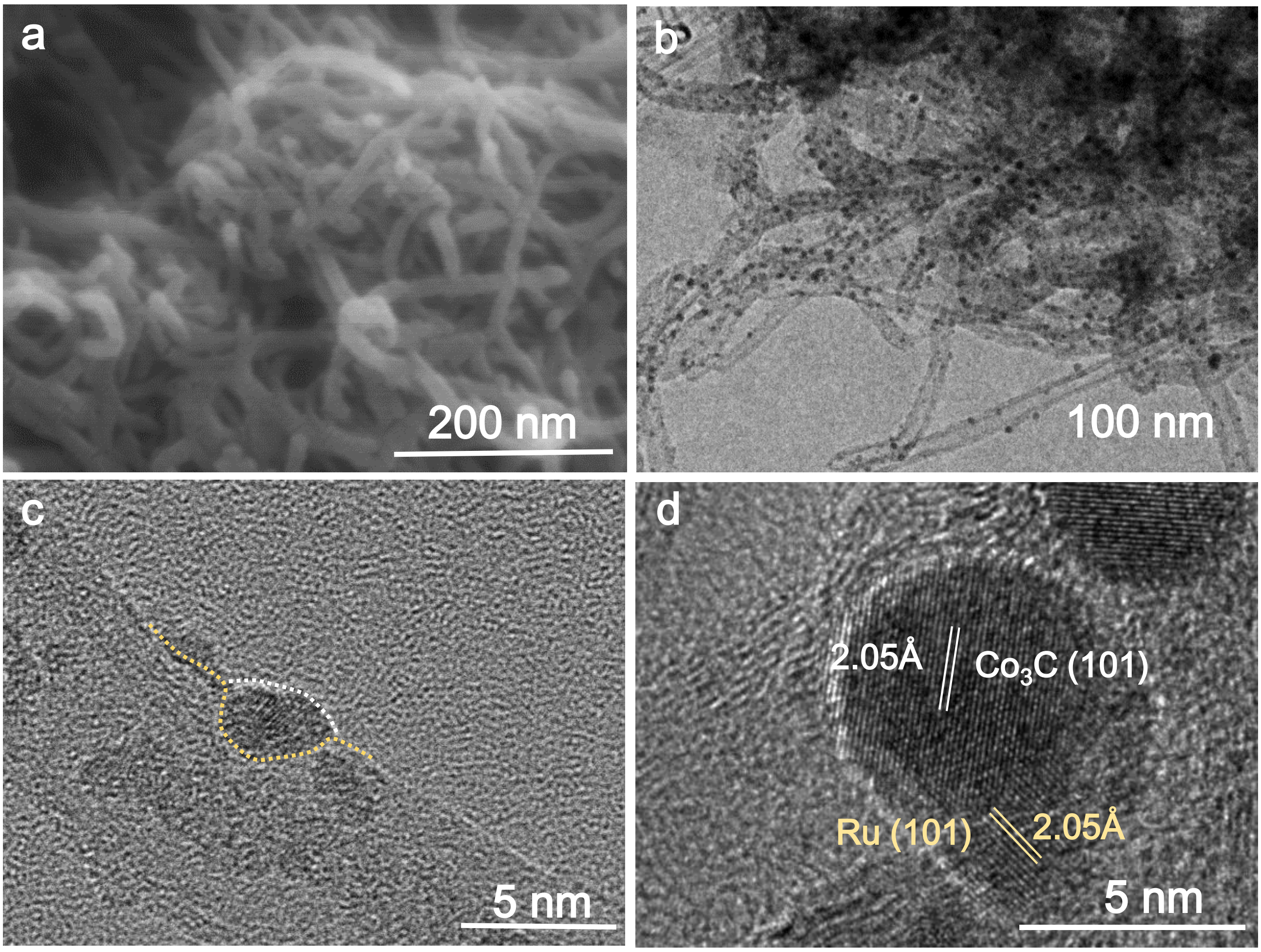

Supplementary Figure 24. (a) SEM, (b)TEM and (c, d) HRTEM images of Ru-Co_3C@CNT.

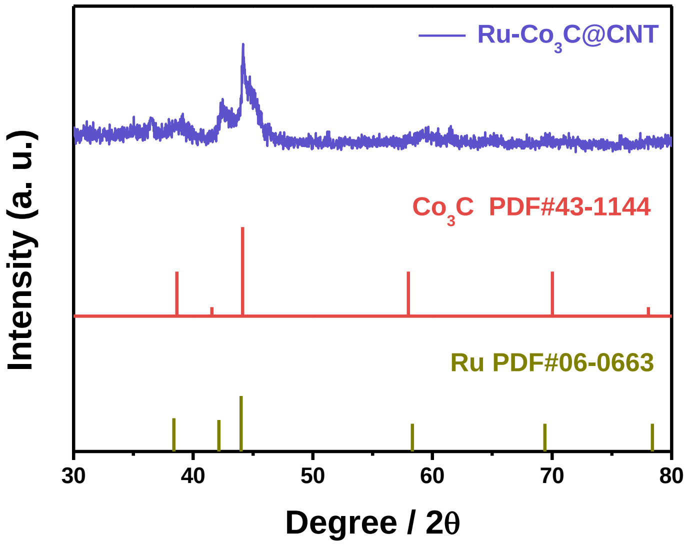

Supplementary Figure 25. XRD pattern of Ru-Co_3C@CNT.

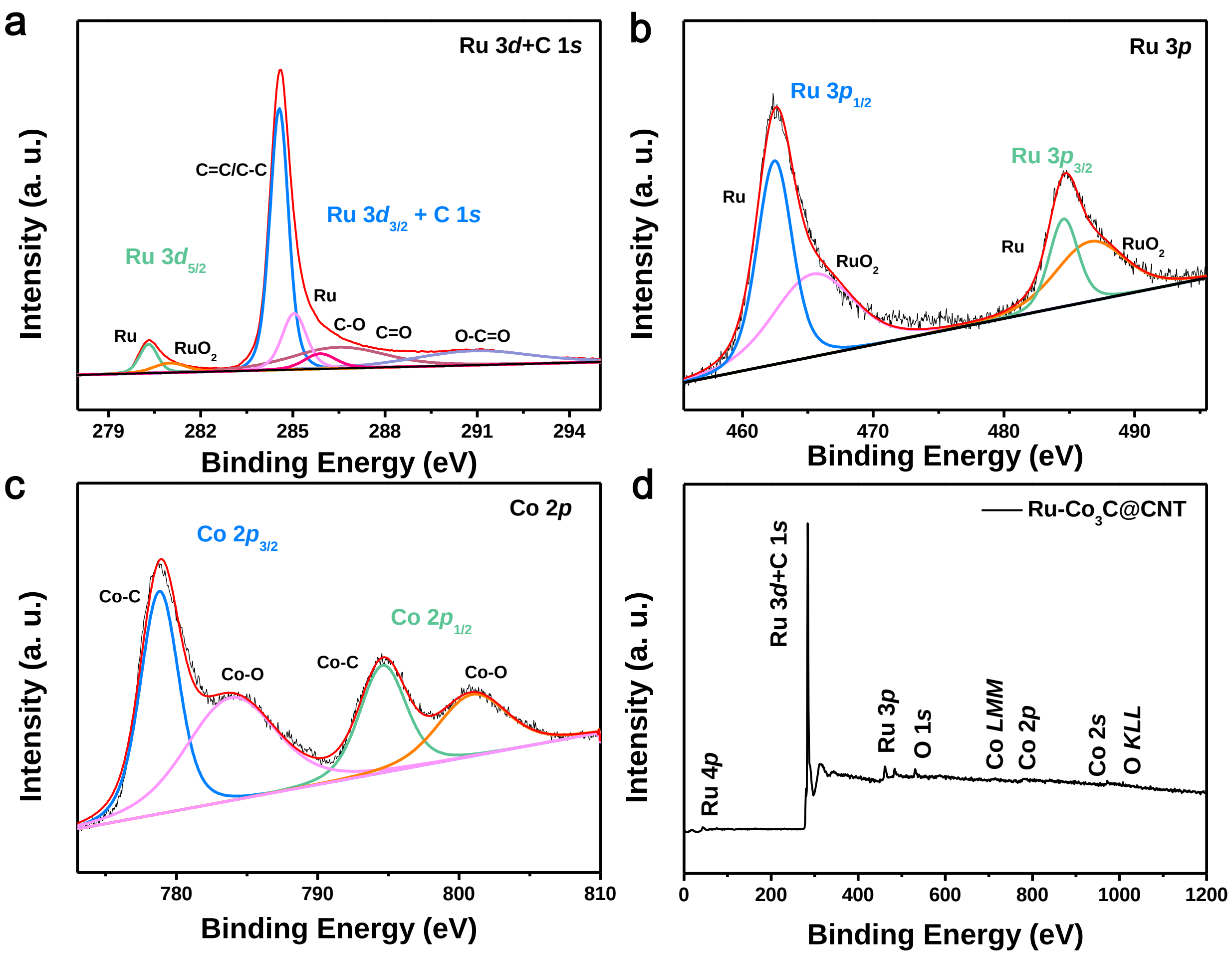

Supplementary Figure 26. XPS spectra of (a) Ru 3*d* + C 1*s*, (b) Ru 3*p*, (c) Co 2*p* and (d) survey spectrum in Ru-Co_3C@CNT.

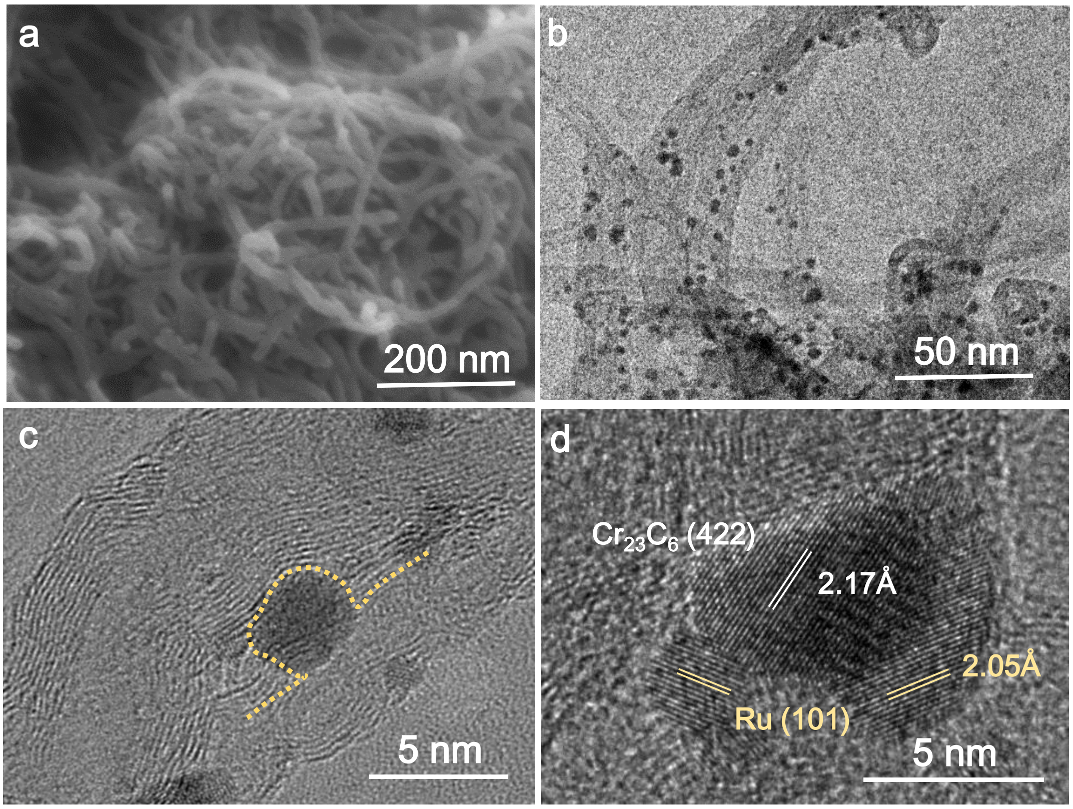

Supplementary Figure 27. (a) SEM, (b) TEM and (c,d) HRTEM images of Ru-$Cr_{23}C_6$@CNT.

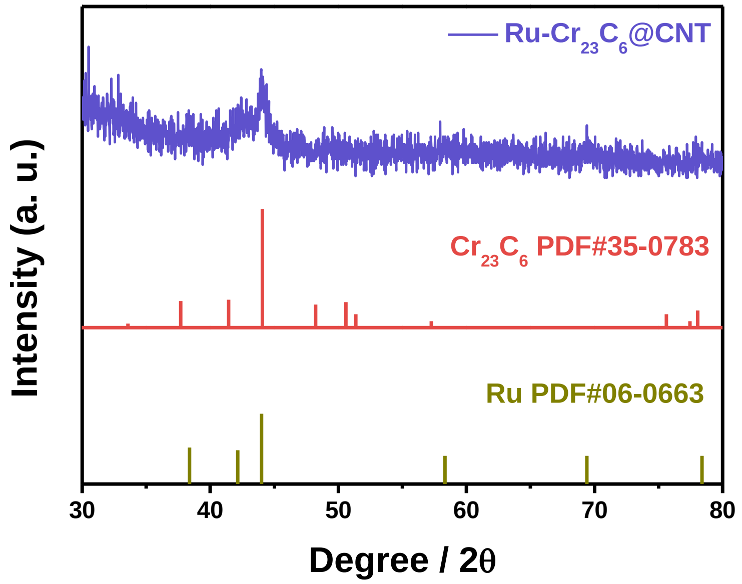

Supplementary Figure 28. XRD pattern of Ru-$Cr_{23}C_6$/CNT.

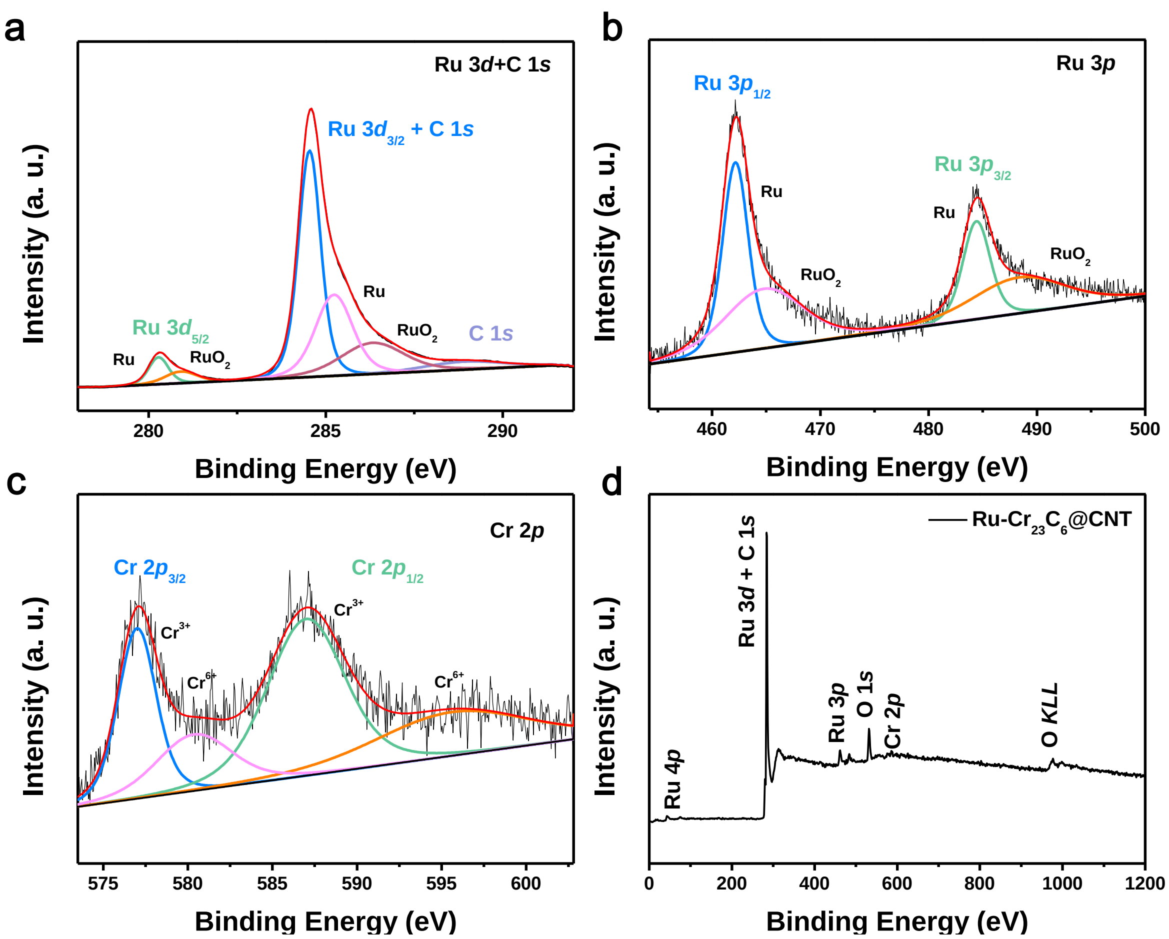

Supplementary Figure 29. XPS spectra of (a) Ru 3*d* + C 1*s*, (b) Ru 3*p*, (c) Cr 2*p* and (d) survey spectrum in Ru-$Cr_{23}C_6$@CNT.

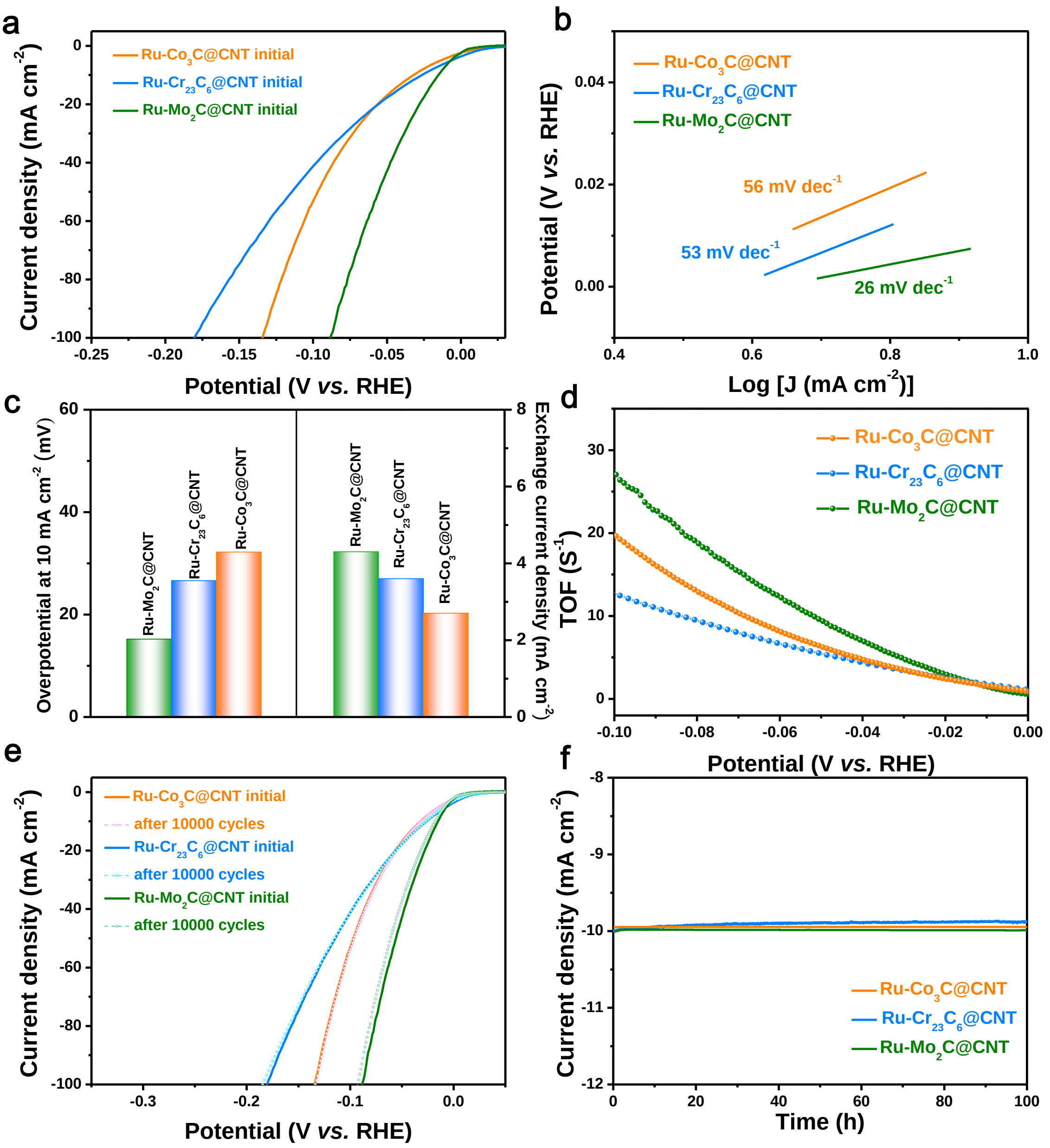

Supplementary Figure 30. Electrocatalytic HER performance test of catalysts in N_2-saturated 1.0 M KOH solution. (a) HER polarization curves of Ru-Co_3C@CNT, Ru-$Cr_{23}C_6$@CNT and Ru-Mo_2C@CNT catalysts. (b) Tafel plots obtained from the polarization curves in (a). (c) Comparison of overpotential changes at 10 mA cm^{-2} and exchange current density. (d) TOF per surface metal site of Ru-$Cr_{23}C_6$@CNT and Ru-Co_3C@CNT and Ru-Mo_2C@CNT catalysts. (e) Polarization curves for Ru-Co_3C@CNT, Ru-$Cr_{23}C_6$@CNT and Ru-Mo_2C@CNT before and after 10000 cycles. (f) Current-time (i-t) stability curves up to 100 h duration of Ru-Co_3C@CNT, Ru-$Cr_{23}C_6$@CNT and Ru-Mo_2C@CNT were recorded in 1.0 M KOH solutions.

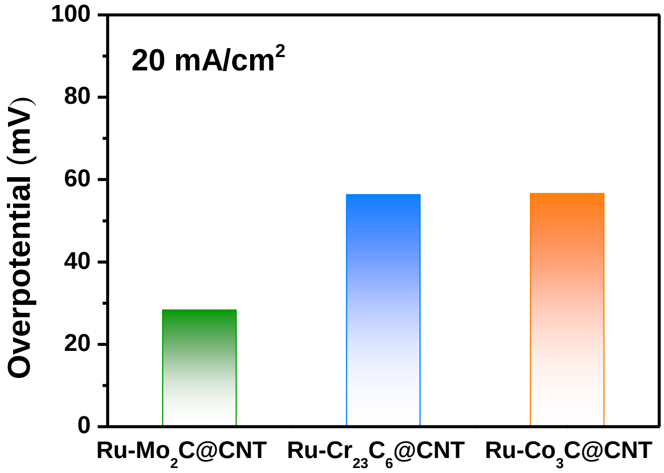

Supplementary Figure 31. Comparison of overpotential changes at 20 mA cm^{-2} of Ru-Mo_2C@CNT, Ru-$Cr_{23}C_6$@CNT and Ru-Co_3C@CNT catalysts.

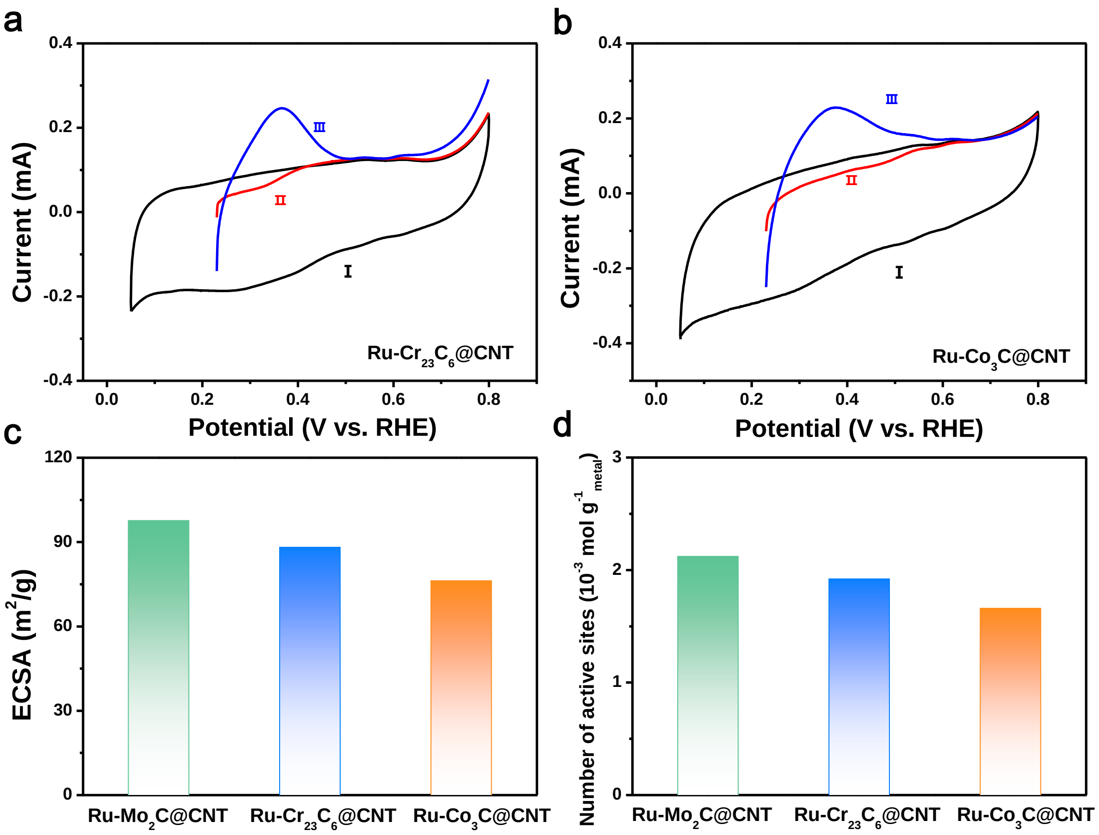

Supplementary Figure 32. Copper UPD in 0.5 M H_2SO_4 in the (I, II) absence and (III) presence of 5 mM $CuSO_4$ on (a) Ru- $Cr_{23}C_6$@CNT and (b) Ru-Co_3C@CNT. For II and III, the electrode was polarized at 0.23 V for 100 s to form the UPD layer. (c)Estimation of the ECSA and (d) active sites using the Cu-UPD method of Ru-Mo_2C@CNT, Ru-$Cr_{23}C_6$@CNT and Ru-Co_3C@CNT.

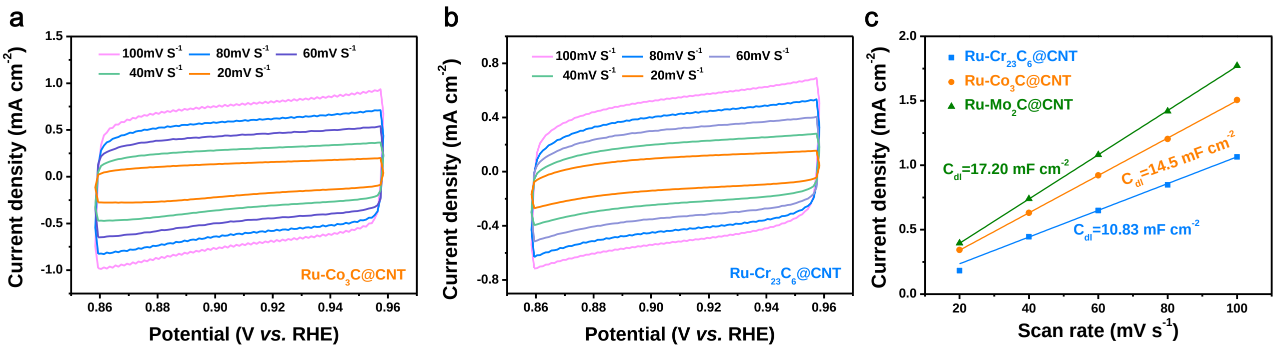

Supplementary Figure 33. CV curves measured at different scan rates from 20 to 100 mV s^{-1} in 1.0 M KOH for (a) Ru-Co_3C@CNT and (b) Ru-$Cr_{23}C_6$@CNT. (c) Capacitive current at middle potential of CV curves as function of scan rates for Ru-Co_3C@CNT, Ru-$Cr_{23}C_6$@CNT and Ru-Mo_2C@CNT.

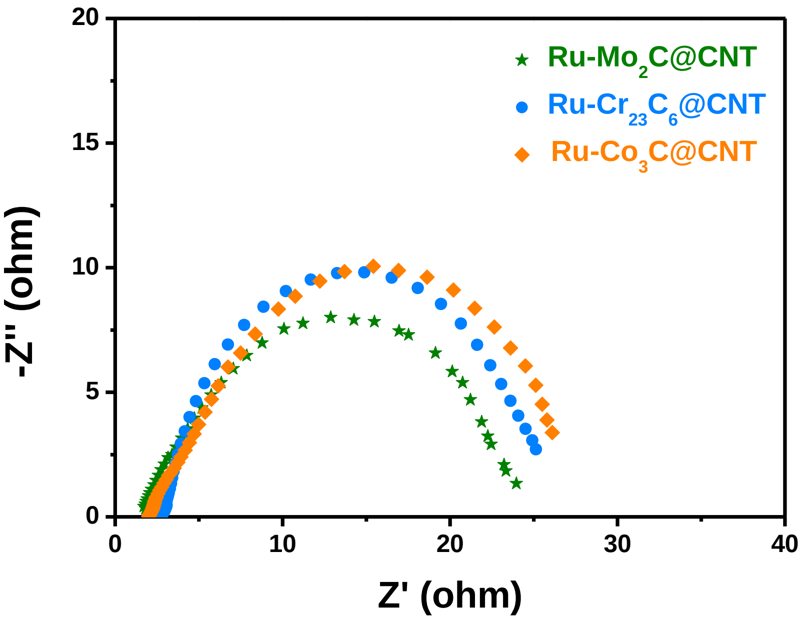

Supplementary Figure 34. EIS Nyquist plots of Ru-Mo_2C@CNT, Ru-$Cr_{23}C_6$@CNT and Ru-Co_3C@CNT catalysts.

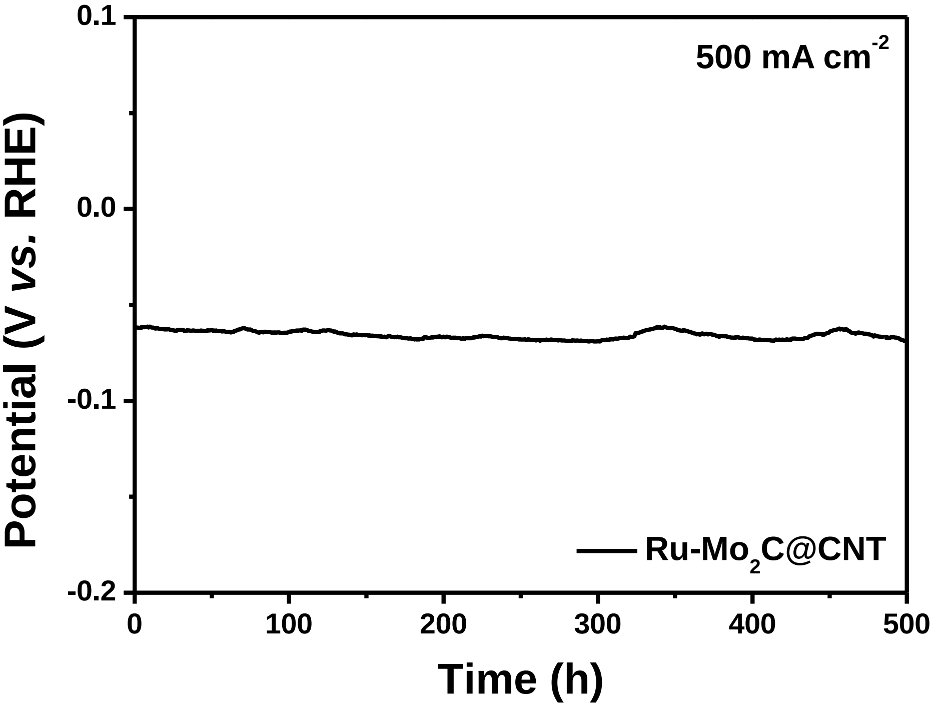

Supplementary Figure 35. The chronopotentiometric curve of the Ru-Mo_2C@CNT electrode tested at a constant current density of 500 mA cm^{-2} for 500 h.

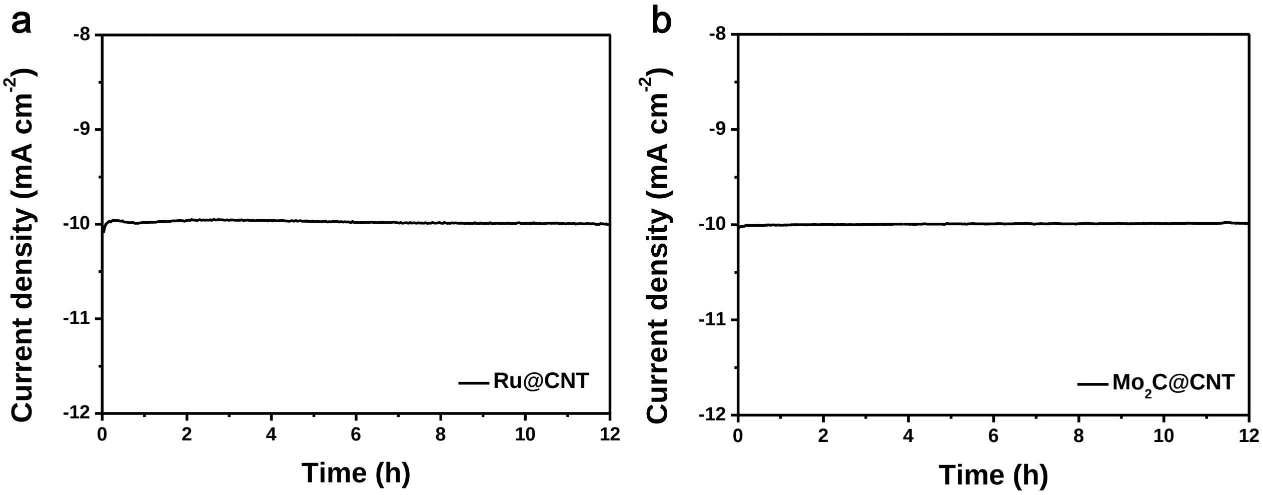

Supplementary Figure 36. (a) Polarization curves for Mo_2C@CNT and Ru@CNT catalysts before and after 10000 cycles. (b, c) i-t curves of Ru@CNT and Mo_2C@CNT under the temporal evolution of the potential required to maintain 10 mA cm^{-2} for 12 h.

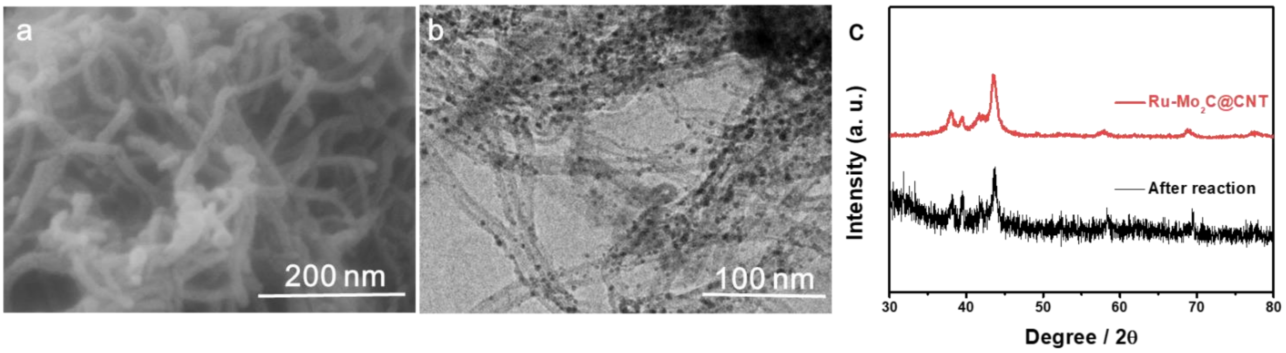

Supplementary Figure 37. (a) SEM image, (b) TEM image and (c) XRD pattern of Ru-Mo_2C@CNT after stability test.

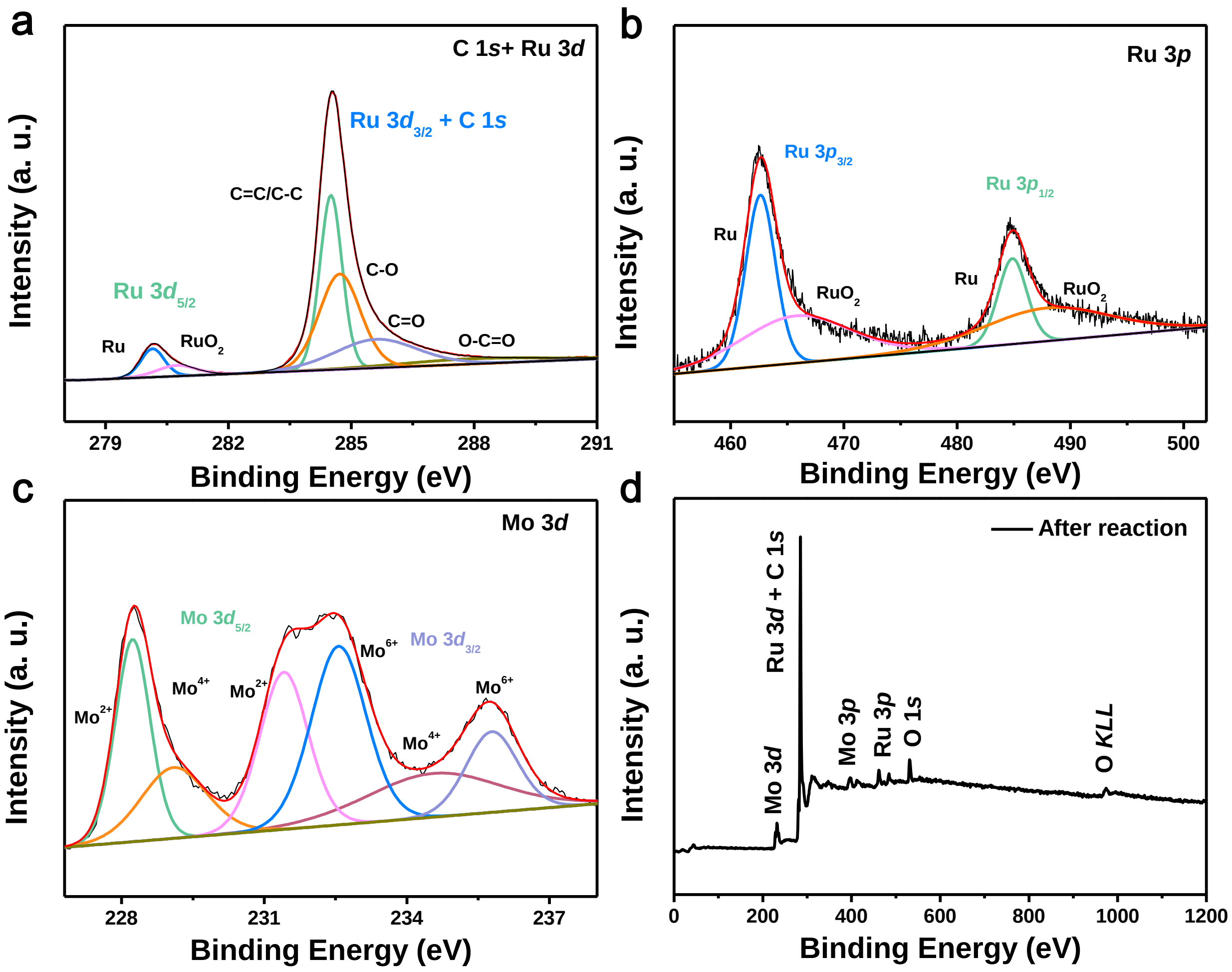

Supplementary Figure 38. XPS spectra of Ru-Mo_2C@CNT after stability test.

Supplementary Table 1. Atomic ratios of Ru-Mo_2C@CNT characterized by ICP.

	Ru atom%	Mo atom%
1:2	44.8	55.2
1:1	61.5	38.5
2:1	76.2	23.8

Supplementary Table 2. The exchange current densities of various samples.

Catalyst	j_0, normalized (mA cm^{-2})	Reference
Ru-Mo_2C@CNT	4.3	This work
Ru-$Cr_{23}C_6$@CNT	3.6	This work
Ru-Co_3C@CNT	2.7	This work
Pt/C	2.2	This work
Ru@CNT	2.0	This work
Mo_2C@CNT	0.28	This work
Ru@C_2N	1.9	Nat. Nanotechnol. 12, 441-446 (2017)
Ru@MWCNT	2.4	Nat. Commun. 11, 1278 (2020)
Pt NPs	2.82	Angew. Chem. Int. Ed. 58, 5432-5437 (2019)
PtNi-O/C	2.18	J. Am. Chem. Soc. 140, 9046-9050 (2018)
Pt (pc)	0.5	ECS Transactions. 50, 2163 (2013)
R-MoS_2@NF	2.27	Adv. Mater. 30, 1707105 (2018)
Mo_2C	0.12	Nat. Commun. 10, 1217 (2019)
C-MoS_2	1.28	Nat. Commun. 10, 1217 (2019)
Pt/MMC	3.2	Adv. Funct. Mater. 29, 1901217 (2019)

Supplementary Table 3. TOF values of Ru-Mo_2C@CNT and reported electrocatalysts in 1.0 M KOH.

Catalyst	Overpotential (V)	TOF (s^{-1})	Reference
Ru-Mo_2C@CNT	0.1	21.9	This work
Ru-Mo_2C@CNT	0.01	1.2	This work
N,P-doped Mo_2C@carbon	0.01	3.71×10^{-3}	ACS Nano 10, 8851-8860 (2016)
L-RP/C	0.02	3.64	Adv. Mater. 30, 1800047 (2018)
Ru@MWCNT	0.025	0.40	Nat. Commun. 11, 1278 (2020)
Ru@C_2N	0.025	0.75	Nat. Nanotechnol. 12, 441-446 (2017)
Co-NiS_2 NSs	0.1	0.55	Angew. Chem. Int. Ed. 58, 18676-18682 (2019)
CoP/Ni_5P_4/CoP	0.1	1.22	Energy Environ. Sci.11, 2246-2252 (2018)
np-$Cu_{53}Ru_{47}$	0.1	1.139	ACS Energy Lett. 5, 192–199 (2020)
$MoNi_4$/MoO_{3-x}	0.1	1.13	Adv. Mater. 29, 1703311 (2017)
Ru@GnP	0.1	0.145	Adv. Mater. 30, 1803676 (2018)
SLNP	0.1	4.2	Adv. Mater. 32, 1908521 (2020)
P-Fe_3O_4/IF	0.15	0.242	Adv. Mater. 31, 1905107 (2019)
o-$CoSe_2$|P	0.2	14.95	Nat. Commun. 9, 2533 (2018)
$Ru_{56}Pd_{44}$/C	0.2	2.9	ACS Cent. Sci. 5, 1991-1997 (2019)
Ru@NC	0.2	10.8	Angew. Chem. 57, 5848 -5852 (2018)
2H $Nb_{1.35}S_2$	0.28	100	Nat. Mater. 18, 1309-1314 (2019)
NiMoN@NiFeN	0.3	0.09	Nat. Commun. 10, 5106 (2019)

Supplementary Table 4. Summary of recently reported representative HER catalysts in 1.0 M KOH.

Catalyst	Overpotential @10 mA cm^{-2}	Tafel slope (mV dec^{-1})	Reference
Ru@CQDs	10	47	Adv. Mater. 30, 1800676 (2018)
Ru_2P/WO_3@NPC	15	18	Angew. Chem. Int. Ed. 60, 4110-4116 (2021)
Ru-Mo_2C@CNT	15	26	This work
Ru@MWCNT	17	27	Nat. Commun. 11, 1278 (2020)
PP-Ru/RuO_2-GC	25	65	ACS Catal. 8, 11094-11102 (2018)
Ru@NC	26	36	Angew. Chem. Int. Ed. 130, 5950-5954 (2018)
RuCo@NC	28	31	Nat. Commun. 8, 14969 (2017)
Ru-MoO_2	29	31	J. Mater. Chem. A. 5, 5475-5485 (2017)
Ru-Ni@Ni_2P-NRs	31	41	J. Am. Chem. Soc. 140, 2731-2734 (2018)
Ru@CN	32	53	Energy Environ. Sci. 11, 800-806(2018)
Ru-NGC	37	40	Chem. Commun. 55, 965-968 (2019)
CNx@Ru/MWCNT	39	28	ChemCatChem 11, 1970-1976 (2019)
Ru@NG-4	40	76	Sustain. Energy Fuels 1, 1028-1033(2017)
Ru ND/C	43.4	49	Chem. Commun. 54, 4613-4616(2018)
CoRu@NC	45	66	Nanotechnology 29, 225403 (2018)
RuP_2@NPC	52	69	Angew. Chem. Int. Ed. 56, 11559-11564 (2017)
ah-RuO_2@C	63	62	Nano Energy 55, 49-58 (2019)
RuPx@NPC	74	70	ChemSusChem 11, 743-752 (2018)
$Cu_{2-x}S$@Ru NPs	82	48	Small 13, 1700052 (2017)
RuO_2-NWs@g-CN	95	70	ACS Appl. Mater. Interfaces 8, 28678-28688 (2016)

Supplementary Table 5. Overpotential and stability values of Ru-Mo_2C@CNT and reported electrocatalysts at 1000 mA cm^{-2} in 1.0 M KOH.

Catalyst	Overpotential at 500 mA cm^{-2} (mV)	Overpotential at 1000 mA cm^{-2} (mV)	Stability (h)	Reference
Ru-Mo_2C@CNT	56	78	1000	This work
Co(10.4)/Se-MoS_2-NF	230	382	350	Nat. Commun. 11, 3315 (2020)
HC-MoS_2/Mo_2C	308	412	24	Nat. Commun. 11, 3724 (2020)
$NiMoO_x$/NiMoS	174	236	50	Nat. Commun. 11, 5462 (2020)
P-Fe_3O_4/IF	210	240	1000	Adv. Mater. 31, 1905107 (2019)
FeIr/NF	125	204	400	Appl. Catal. B: Environ. 278, 119327 (2020)
Co-Mo_5N_6	200	280	10	Adv. Energy. Mater. 10, 2002176 (2020)
IrFe/NC	430	850	12	Appl. Catal. B: Environ. 258, 117965 (2019)
Ni_2P/NF	200	306	2.7	J. Am. Chem. Soc. 141, 7537-7543 (2019)
FeP/Ni_2P	220	293	40	Nat. Commun. 9, 2551 (2018)
$MoNi_4$/MoO_{3-x}	114	155	20	Adv. Mater. 29, 1703311 (2017)
NC/Ni_3Mo_3N/NF	480	680	50	Appl. Catal. B: Environ. 272, 118956 (2020)
